# Positive regulation of PFKFB3 by PIM2 promotes glycolysis and paclitaxel resistance in breast cancer

**DOI:** 10.1002/ctm2.400

**Published:** 2021-05-01

**Authors:** Chao Lu, Pengyun Qiao, Yonghong Sun, Chune Ren, Zhenhai Yu

**Affiliations:** ^1^ Department of Reproductive Medicine Affiliated Hospital of Weifang Medical University Weifang Shandong Province P. R. China; ^2^ Department of Pathology Affiliated Hospital of Weifang Medical University Weifang Shandong Province P. R. China

**Keywords:** glycolysis, PFKFB3, PIM2, protein modification, resistance

## Abstract

**Background:**

Breast cancer (BC) is one of the most common female malignancies in the world. Chemotherapeutic resistance is the major cause of BC therapy failure, leading to tumor recurrence and metastasis. Studies have illustrated the close relationship between glycolysis and BC progression and drug resistance. The key glycolysis regulator, PFKFB3 makes a difference during BC progression and drug resistance. However, the mechanism remains to be unknown.

**Methods:**

Mass spectrometry analyses were used to found that PIM2 was a potential new binding protein of PFKFB3. Co‐immunoprecipitated and western blot were used to verify the interaction between PIM2 and PFKFB3 in BC and the molecular mechanism by which PIM2 phosphorylates PFKFB3 in regulating the protein function. PFKFB3 mutant forms were used to demonstrate the need for PFKFB3 in BC drug resistance.

**Results:**

We identified that PIM2 is a new binding protein of PFKFB3. We used biochemical methods to determine that PIM2 can directly bind and change the phosphorylation of PFKFB3 at Ser478 to enhance PFKFB3 protein stability through the ubiquitin‐proteasome pathway. Importantly, phosphorylation of PFKFB3 at Ser478 promoted glycolysis, BC cell growth, and paclitaxel resistance together with PIM2 in vitro and in vivo.

**Conclusion:**

Our study demonstrates that PIM2 mediates PFKFB3 phosphorylation thus regulates glycolysis and paclitaxel resistance to promote tumor progression in BC and provides preclinical evidence for targeting PFKFB3 as a new strategy in BC treatment to battle paclitaxel resistance.

## Graphical abstract

We demonstrates that PIM2 mediates PFKFB3 phosphorylation thus regulates glycolysis and paclitaxel resistance to promote tumor progression in breast cancer, and provides preclinical evidence for targeting PFKFB3 as a new strategy in breast cancer treatment to battle paclitaxel resistance.

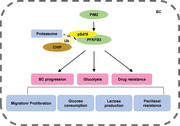

## 1 BACKGROUND

Breast cancer (BC) is one of the most common female malignancies in the world. The high morbidity and mortality of BC make this a serious and life‐threatening condition for women.^1^, ^2^ However, the mechanism of BC development remains unclear.

Cancer cells prefer glycolysis to provide the energy rather than oxidative phosphorylation even with sufficient oxygen and normal mitochondrial function; the phenomenon, historically recognized as "Warburg Effect" is universal in BC.^3^, ^4^ As the malignancy of the tumor increases, glycolytic activity gradually increases. Glycolysis flux in tumors is regulated by different enzymatic reactions.^5^ 6‐phosphofructose‐2‐kinase/fructose‐2,6‐bisphosphatase 3 (PFKFB3), a key regulator of glycolysis, plays an indispensable regulatory role in BC glycolysis and malignant progression.^6^, ^7^ PFKFB3 belongs to the PFK‐2/FBPase (PFKFB) family function as homodimeric and bifunctional enzymes. Comparing kinase activity among four family members (PFKFB1‐4) to shunt glucose toward glycolysis, PFKFB3 is the highest.^8^ Progesterone and estradiol receptors in BC can bind to response elements in the promoter region of *PFKFB3* to induce PFKFB3 expression to stimulate glucose uptake and glycolysis of tumors.^9^ Silencing the expression of PFKFB3 in BC can reduce the glycolytic flux, inhibit proliferation of cancer cells, and induce tumor cell apoptosis.^8^, ^10^ Additionally, related research shows that cancer cells with PFKFB3 modificated post‐translationally, such as phosphorylation, acetylation, and methylation, can better accommodate to glucose metabolism in different stress conditions. Ser461 phosphorylation of PFKFB3, mediated by AMP‐activated protein kinase in BC, can rapidly provide adenosine triphosphate (ATP) to prevent cell death due to mitotic arrest.^7^ The acetylation of PFKFB3 can affect its ability to shuttle between the nucleus and cytoplasm.^11^ Heme oxygenase 1 and carbon monoxide can induce PFKFB3 hypomethylation and polyubiquitination to help cancer cells resist oxidative stress.^12^ Although the regulatory role of post‐translational modification of PFKFB3 in tumor regulation is of great importance, the mechanism of PFKFB3 protein stability in BC is still unclear.

Proviral insertion in murine lymphomas 2 (PIM2) belongs to the proto‐oncogene family of serine/threonine kinases (PIM1, PIM2, PIM3), whose protein structure is highly conserved suggesting that it has an important biological function.^3^ Our previous research has found that PIM2 plays an essential role in BC.^13^ PIM2 lacks an independent regulatory domain and has a unique catalytic structure, which facilitates the design of targeted small molecule inhibitors.^14^ The JAK/STAT pathway, NF‐κB, and HSP90 regulate the activity of PIM2 primarily.^15^ PIM2 participates in the cell cycle, metabolism, proliferation, differentiation, and other biological processes to promote tumorigenesis by targeting key factors in different signaling pathways.^16^ Our previous study found that PIM2 is abnormally highly expressed in BC. PIM2 could also phosphorylate FBP1 on Ser144, increase PD‐L1 expression regulated by p65, and promote BC growth in vitro and in vivo.^13^ PIM2 could also decrease Tristetraprolin through the ubiquitin‐proteasome pathway to promote BC proliferation and migration.^17^ We also confirmed that PIM2 was highly expressed in BC paclitaxel‐resistant strains, and its inhibitor SMI‐4a could increase the sensitivity of BC paclitaxel chemotherapy.^3^ Above all, PIM2 plays an indispensable regulatory role in the progression of BC; however, its potential molecular mechanism needs to be further explored.

**HIGHLIGHTS**

- PIM2 could directly bind and change the phosphorylation of PFKFB3 at Ser478.
- PIM2 enhanced PFKFB3 protein stability through the ubiquitin‐proteasome pathway.
- Phosphorylation of PFKFB3 at Ser478 promoted glycolysis and was required for breast cancer cell growth in vitro and in vivo.
- PIM2 and PFKFB3 enhanced paclitaxel resistance in vitro and in vivo.

Here, we identified PIM2 as a new binding protein of PFKFB3. We utilized biochemical methods to determine that PIM2 can directly bind and change the phosphorylation of PFKFB3 at Ser478 to enhance PFKFB3 protein stability through the ubiquitin‐proteasome pathway. Importantly, phosphorylation of PFKFB3 at Ser478 promoted glycolysis, BC cell growth, and paclitaxel resistance together with PIM2 in vitro and in vivo. Therefore, our research clarifies the regulatory characteristics of BC glucose metabolism and provides theoretical support for new methods for targeted diagnosis and treatment of tumors.

## 2 MATERIALS AND METHODS

### 2.1 Cell culture and transfection

HEK293T, MDA‐MB‐231 and MCF7 and cells were purchased from American‐type culture collection (ATCC). Paclitaxel‐resistant MCF‐7 cells were derived from our previous screening. All cell lines were cultured in DMEM medium (HyClone) supplemented with 10% fetal bovine serum (Gibco) and 100 μg/ml penicillin/streptomycin at 37℃ in 5% CO2 (v/v). Plasmid transfection was performed as described previously.^13^

#### 2.1.1 Primary mouse embryonic fibroblast

The uteri at about 13.5 days of gestation were obtained, and the embryos were taken out. The head and internal organs were cut off, and the remaining body parts were washed with PBS. Bodies completely cut into pieces by scissors. The cells were digested by trypsin in a 37°C incubator for 5–10 min. The digested cells were suspended in DMEM medium and then spread evenly into the culture dish at 37℃ in 5% CO2 (v/v).

### 2.2 Plasmids

The plasmids were constructed by Gene Pharma. Full‐length complementary DNA (cDNA) of related genes (PIM2, PFKFB3, CHIP) was cloned into basic plasmids with tags such as /HA, Flag, pFlag‐CMV4, GFP, His, pGEX‐4T‐1, and so on using overlap PCR method to obtain point mutation fragments. The shRNA sequence was provided in Table S1.

### 2.3 Antibodies

Antibodies against β‐actin (60008‐1‐Ig, 1:5000), HA tag Rabbit (51064‐2‐AP, 1:3000), HA Tag Mouse (66006‐2‐Ig, 1:5000), GFP tag Rabbit (50430‐2‐AP, 1:3000), Flag tag Rabbit (20543‐1‐AP, 1:3000), and Flag Tag Mouse (66008‐3‐Ig, 1:3000), were purchased from Proteintech. Antibodies against PFKFB3 (SAB1410957, 1:3000), PIM2 Rabbit (HPA000285, 1:3000), PIM2 Mouse (SAB1407095, 1:3000), and CHIP (SAB1300354, 1:500) were obtained from Sigma. Mouse and rabbit IgG antibodies were purchased from Santa Cruz Biotechnology.

The phosphorylated antibody on PFKFB3‐Ser478 site (pS478, 1:500) was produced by Shanghai Genomic Inc.

### 2.4 Immunoprecipitation, GST pull‐down assay, and western blot

The cells were processed according to different experimental requirements, and then the cells were lysed by protein lysis extract buffer consisting of 0.5% NP40, 150 mM NaCl, 1 mM EDTA, and 50 mM Tris‐HCl (pH 7.4). Cell lysate was centrifuged at 13000 × g at 4°C for 15 min, and the supernatant was collected. Note that 70 μl of supernatant was taken out as input.  The supernatant was incubated with indicated antibodies and protein‐A‐agarose overnight at 4℃. Isotype‐matched IgG was used as negative control. After the beads were washed 3–5 times with ice‐cold NP‐40 buffer, beads were decoupled by sodium dodecyl sulfate (SDS) loading buffer boiling. The samples were analyzed by western blot. GST pull‐down assay was performed as described previously.^3^, ^18^

The proteins separated by SDS‐PAGE and transferred to polyvinylidene fluoride (PVDF) membranes. Prepared primary and secondary antibodies were incubated after blocking with 5% milk blocking solution dissolved in Tris‐Buffered Saline Tween‐20 (TBST) with the components including 0.1% Tween20, 135 mM NaCl, and 20 mM Tris.

### 2.5 Confocal microscopy analyses

Confocal microscopy analyses were performed as described previously.^13^

### 2.6 Stable cell lines

To construct PFKFB3 knockdown and rescue stable cells, ShPFKFB3 was cloned into the pGLVH1 vectors, and lentiviral pLVX‐IRES‐Neo vector embedded fragments with PFKFB3 wild‐type (WT) and mutants (S478A, S478D). Stable PFKFB3 knockdown was constructed and screened with puromycin. HA‐tagged rPFKFB3 was selected by G418, and single cell was expanded.

### 2.7 Cell glucose, lactate, and PFKFB3 activity measurement

Glucose level was detected by glucose (GO) assay kit (Sigma, GAGO20), and Lactate and PFKFB3 activity measurement were quantified using Kit (Bio Vision, catalog K627, K776). Cells were divided into six‐well plates and pre‐treated according to experimental requirements.

### 2.8 Clone formation, wound healing assay, and cell proliferation analysis

Clone formation: Cells (200–500 cells/plate) were evenly spread in a six‐well plate and cultured for 2 weeks. Then the cells were performed as described previously.^3^ The Image J software was used to quantify distances/areas in scratch assays

Wound healing assay: A straight line was drawn into the six‐well plates which overgrown with cell. Taken pictures 1 day later.

Cell proliferation analysis: Cells (15000–2000cells/plate) were cultured in 24‐well plates. Continuous cell count for 3 days by cell counter. GraphPad Prism 5.0 software was used to data processing.

### 2.9 Immunohistochemistry

The animal experiments and human tissue specimens involved in the research were approved by the Ethics Committee of Weifang Medical University and the Affiliated Hospital of Weifang Medical College. The animal ethics number was 2020SDL033. The medical ethics number was 2020YX007. All patients signed informed consent. Our study adhered to the declaration of Helsinki. The expression of PFKFB3 and PIM2 in BC tissues was detected as described previously.^3^ Additionally, Ki67 of mouse tumors from each group was tested as described previously.^2^

### 2.10 Xenografts

Enough rPFKFB3(WT, S478A or S478D) stable expression MCF‐7 cells (5 × 10^6^/100μL) were prepared and injected subcutaneously into female 4‐week‐old BALB/c nude mice. The experimenter regularly measured the tumor volume for 3 weeks. When the experimental period was over, tumor size (diameter) exceeded 15 mm, or the mouse had health problems, the mouse would be killed.

For paclitaxel‐resistant experiments, sufficient MCF‐7/TaxR cells were prepared. Tumor formation experiment was performed on 4‐week‐old female BALB/c nude mice. The mice were then randomly assigned to four groups: PBS (control), paclitaxel (10 mg/kg) alone, PFK15 (25 mg/kg) alone, and PFK15 combined with paclitaxel after 1 week. According to the group, intraperitoneal injection was given every 3 days, and tumor volume was measured regularly. After 2 weeks treatment or tumor size (diameter) exceeded 15 mm, the mice were sacrificed, and the body and tumor weights were measured. When the mouse had health problems, the mouse also would be killed. Volume calculation and experimental treatment of tumor tissue were performed as described previously.^3^

### 2.11 Statistical analysis

All results were analyzed and graphed by GraphPad Prism 7 (GraphPad Software). All data shown represent the results obtained from at least three biological or technical replicates as indicated with standard deviation of the mean (mean ± sd.). Pearson correlation analysis was used to evaluate the relationship between two variables. Pairwise comparisons were performed using a two‐tailed Student *t* test. *p* values less than 0.05 were considered significant.

## 3 RESULTS

### 3.1 PIM2 is a novel binding partner of PFKFB3

**FIGURE 1:**
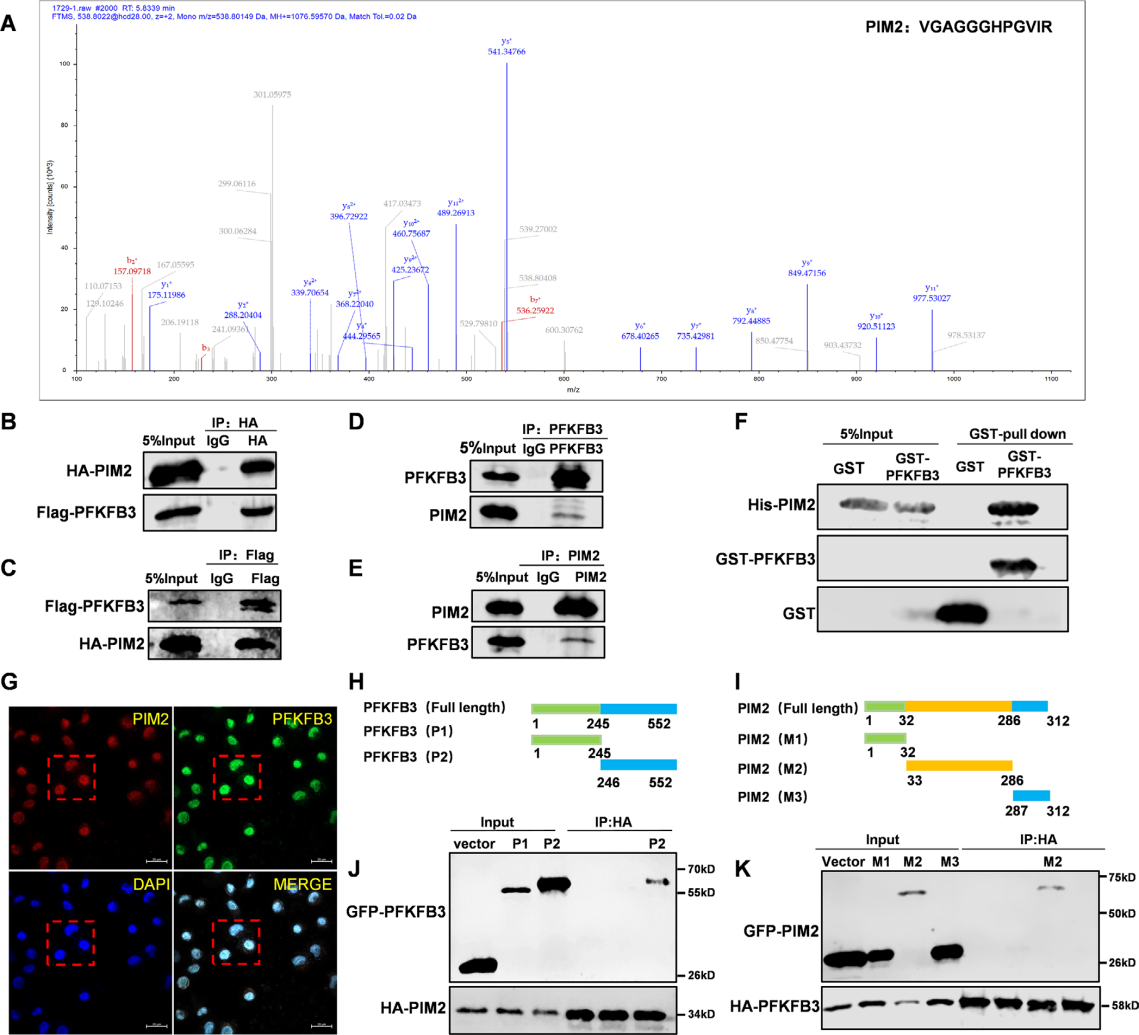
PIM2 interacts with PFKFB3. (A) Mass spectrometry analyses of the immunoprecipitated PFKFB3 complex in MCF‐7 cells. (B, C, D, and E) Immunoprecipitation (IP) and immunoblotting (IB) analyses were performed with indicated antibodies. HA‐(B), Flag‐(C), PFKFB3(D), PIM2(E). (F) GST‐PFKFB3 proteins were pulled down using streptavidin agarose beads induced by His‐PIM2. (G) Confocal immunofluorescence microscopy was performed to analyze localization of PIM2 and PFKFB3 in MCF‐7 cells. Red box mark was partially enlarged in the Figure S1A. (H and I) Schematic representation of PFKFB3(H) and PIM2(I) truncation mutants. (J and K) HEK293T cells were cotransfected with indicated plasmids. Immunoprecipitation with anti‐HA antibody was performed. All experiments were repeated at least 3 times

PFKFB3, a crucial regulator of glycolysis, shows great importance in tumor development.^7^, ^19^, ^20^ To clarify what PFKFB3 functions in BC, we analyzed immunoprecipitated PFKFB3 complex in MCF‐7 cells with spectrometry, finding PIM2 may be a new binding protein of PFKFB3 (Figure 1A and Excel S1). To verify this, we exogenously expressed HA‐PIM2 and Flag‐PFKFB3 in 293T cells and found that PIM2 bound to PFKFB3 through co‐immunoprecipitation (co‐IP) (Figures 1B and 1C). We also performed co‐IP to detect the binding of endogenous PIM2 and PFKB3 in MCF‐7 cells (Figure 1D and E). Moreover, GST‐tagged PFKFB3 could pulldown His‐tagged PIM2, suggesting the direct interaction between PIM2 and PFKFB3 (Figure 1F). We also identified overlapping regions in the expression of PIM2 and PFKFB3 in MCF‐7 cells (Figure 1G and Figure S1A). In order to further identify where PIM2 and PFKFB bind, we divided them into different fragments and performed co‐IP assays. PFKFB3 was divided into two parts: GFP‐tagged P1‐PFKFB3 (amino acid residues 1–245) and GFP‐tagged P2‐PFKFB3 (amino acid residues 246–552) (Figure 1H). PIM2 was divided into three parts: GFP‐tagged M1‐PIM2 (amino acid residues 1–32), M2 (amino acid residues 33–286), and GFP‐tagged M3‐PIM2 (amino acid residues 287–212) (Figure 1I). As shown in Figures 1J and 1K, PIM2 strongly binds to the P2 domain of PFKFB3, and PFKFB3 specifically interacts with the kinase P2 domain. Together, our results demonstrate that PIM2 is a novel binding partner of PFKFB3.

### 3.2 PIM2 phosphorylates PFKFB3 at Ser478

**FIGURE 2:**
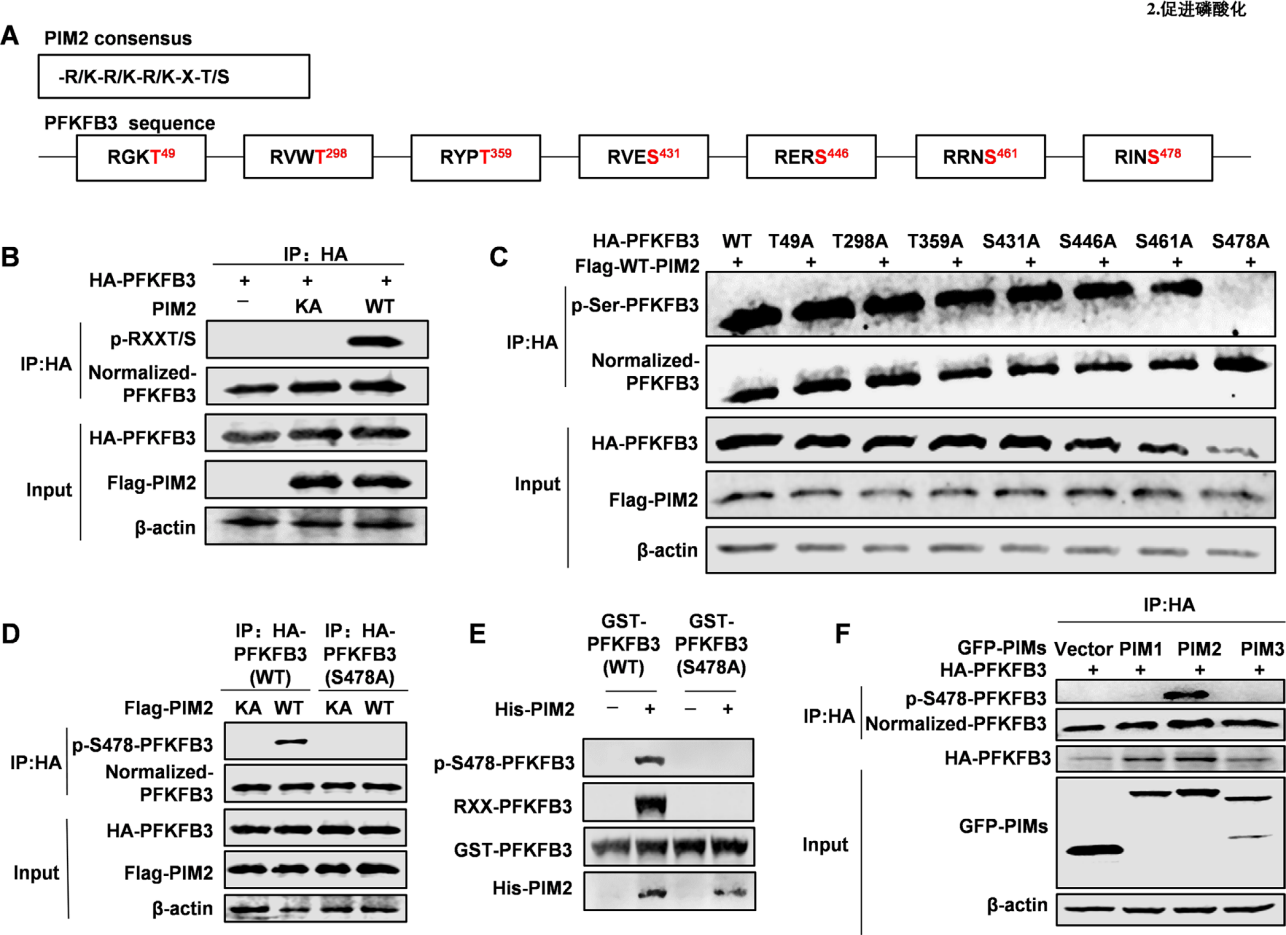
PIM2 phosphorylates PFKFB3 at Ser478. (A) Schematic diagram of sites in PFKFB3 that may be modified by PIM2. (B) HEK293T cells were overexpressed the indicated HA‐PFKFB3 and Flag‐PIM2 (WT or KA) proteins. Immunoprecipitation with an anti‐HA antibody was performed. (C) HEK293T cells were overexpressed the indicated HA‐PFKFB3 (WT or mutant) and Flag‐PIM2 (WT). Immunoprecipitation with an anti‐HA antibody was performed. (D) HEK293T cells were cotransfected with HA‐PFKFB3 (WT or S478A) and Flag‐PIM2 (WT or KA) proteins. Immunoprecipitation with an anti‐HA antibody was performed, followed by Western blot with indicated antibodies. (E) Purified GST‐PFKFB3 was mixed with the indicated bacterially purified His‐PIM2 proteins. An in vitro kinase assay was performed. (F) HEK293T cells were overexpressed the indicated HA‐PFKFB3 and GFP‐PIMs (PIM1, PIM2, PIM3). Immunoprecipitation with an anti‐HA antibody was performed. All experiments were repeated at least 3 times

Because PIM2 is a kinase, and changing the levels of substrate phosphorylation is one of the ways to promote tumorigenesis and development,^18^ we next investigated whether PIM2 can phosphorylate PFKFB3. After analyzing the protein sequence of PFKFB3, we determined that PFKFB3 has 7 ‐R/K‐R/K‐R/K‐X‐T/S conserved sequences specifically recognized by PIM2, suggesting that PIM2 may phosphorylate PFKFB3 (Figure 2A). To verify the hypothesis, we overexpressed Flag‐tagged PIM2 (kinase‐inactive [K61A] or wild‐type [WT]) with HA‐tagged PFKFB3 in 293T cells. It turned out to be that WT PIM2 increased the phosphorylation level of PFKFB3 compared with the control vector or kinase‐inactive PIM2 (Figure 2B). To further determine the specific phosphorylation site, we generated point mutations in all potential phosphorylation sites. As shown in Figure 2C, mutation of position 478 prevented phosphorylation, indicating that PFKFB3 Ser478 contributed to PIM2‐induced phosphorylation. Furthermore, we took advantage of the protein post‐translational modifications database (https://www.phosphosite.org/) and identified phosphorylation of PFKFB3 at Ser478 by proteomic analyses. Next, we generated an antibody to specifically target PFKFB3 Ser478 phosphorylation and determined that PIM2 could not phosphorylate mutated PFKFB3 (Ser478A) (Figure 2D). In addition, we verified that PIM2 could phosphorylate PFKFB3 at Ser478 directly by in vitro kinase assay and the only one to phosphorylates PFKFB3 out of the PIM family (Figure 2E). In summary, our results suggest that Ser478 of PFKFB3 is phosphorylated by PIM2.

### 3.3 PIM2 regulates PFKFB3 protein stability

**FIGURE 3:**
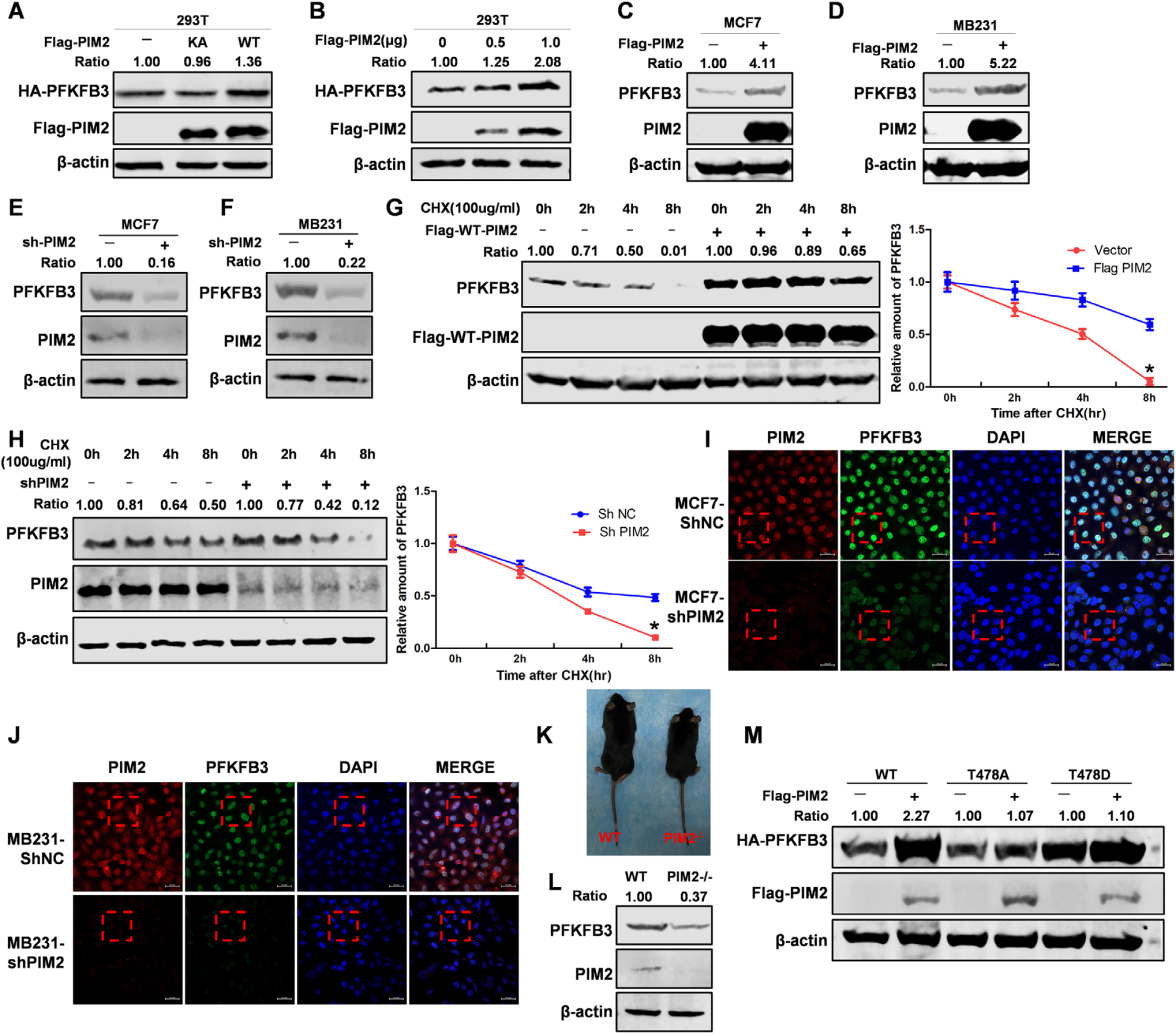
PIM2 regulates PFKFB3 protein stability. (A) HEK293T cells were cotransfected with HA‐PFKFB3 and Flag‐PIM2 (WT or KA) plasmid. Western blot analysis tested whole‐cell lysate after 72‐h transfection. (B) HEK293T cells were cotransfected with HA‐PFKFB3 and Flag‐PIM2 (0, 0.5, 1.0 μg) plasmid. Western blot analysis tested whole‐cell lysate after 72‐h transfection. (C and D) MCF7 or MB231 cells were cotransfected with Flag‐PIM2 plasmid. Whole‐cell lysate after 72‐h transfection. (E and F) MCF7 or MB231 cells were knocked down PIM2 with shRNA. Total cell lysates were prepared. (G) MCF7 cells were transfected with HA‐PFKFB3 and Flag‐PIM2 plasmids, and then treated with CHX for indicated time. Total cell lysates were prepared. (H) MCF‐7 cells with stable knockdown PIM2 proteins were treated with CHX for indicated time. Total cell lysates were prepared. (I and J) MCF7 or MB231 cells were knocked down PIM2 with shRNA. Confocal immunofluorescence microscopy was performed to observe the expression of PIM2 and PFKFB3. Red box mark was partially enlarged in the Figures S1B and S1C. (K) PIM2 gene knockout mice constructed and screened. (L) Detection of PIM2 and PFKFB3 expression in PIM2 knockout mice by WB. (M) MCF‐7 cells were overexpressed the indicated both Flag‐PIM2 and HA‐PFKFB3 (WT, S478A, or S478D) proteins. Total cell lysates were prepared. All experiments were repeated at least 3 times

To further explore the effect of PIM2‐mediated PFKFB3 phosphorylation, we overexpressed Flag‐tagged PIM2 (K61A or WT) with HA‐tagged PFKFB3 in 293T cells. We found that overexpression of WT PIM2 promoted the level of PFKFB3; however, K61A PIM2 expression did not impact PFKFB3 protein levels (Figure 3A). The protein stability of PFKFB3 was dose‐dependent with PIM2 (Figure 3B). Consistently, PIM2 enhanced PFKFB3 protein level in BC cells (Figure 3C and D). Simultaneously, PFKFB3 protein levels decreased in response to shPIM2 knockdown of PIM2 expression in BC cells (Figure 3E and F). To further verify the relationship between PIM2 and PFKFB3 protein stability in BC, Flag‐tagged PIM2 was overexpressed in MCF‐7 cells and treated with cycloheximide, a protein synthesis inhibitor, for various periods of time. PIM2 blocked the degradation of PFKFB3 (Figure 3G). PFKFB3 degradation was significantly accelerated in PIM2 knock down by shPIM2 in MCF‐7 (Figure 3H). Consistently, confocal imaging confirmed that PFKFB3 protein levels were decreased in response to PIM2 knock down in BC cells (Figures 3I and J and Figures S1B and S1C). We previously constructed the PIM2 knockout mouse model^13^ (Figure 3K) and determined that PFKFB3 protein stability was greatly reduced in mouse embryonic fibroblasts (Figure 3L). To verify whether the Ser478 site impacts protein stability, mutant HA‐tagged PFKFB3 (S478A or S478D) was generated. The meaning of S478D is continuous phosphorylation at position Ser478. As it is shown HA‐PFKFB3 MUT (S478A, S478D) protein stability was not significantly changed while HA‐PFKFB3 WT protein level was enhanced with PIM2 expression (Figure 3 M). We conclude that PIM2 regulates PFKFB3 protein stability on the Ser478 site via a CMA pathway.

### 3.4 PIM2 promotes PFKFB3 protein stability via the CHIP‐mediated ubiquitin‐proteasome pathway

**FIGURE 4:**
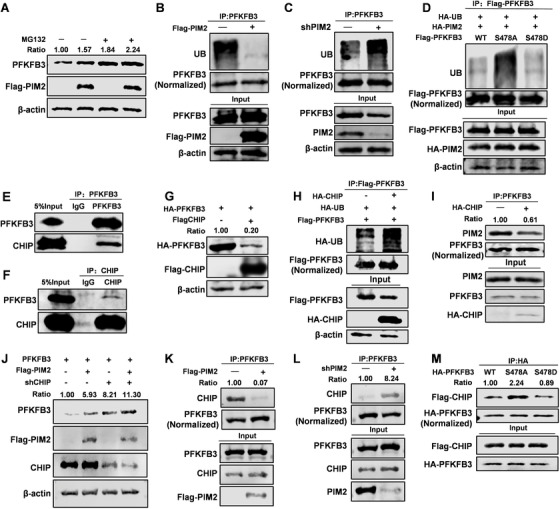
PIM2 promotes PFKFB3 protein stability via the CHIP‐mediated ubiquitin proteasome pathway. (A) MCF7 cells were cotransfected with Flag‐PIM2, and HA‐PFKFB3 plasmids were treated with or without MG132 for 8 h. Total cell lysates were prepared. (B and C) MCF7 cells were cotransfected with Flag‐PIM2 or sh‐PIM2. Immunoprecipitation with an anti‐PFKFB3 antibody was performed. (D) MCF7 cells were co‐overexpressed the indicated HA‐PIM2 and Flag‐PFKFB3 (WT, S478A or S478D) proteins. Immunoprecipitation with an anti‐Flag antibody was performed. (E and F) Immunoprecipitation (IP) and immunoblotting (IB) analyses were performed with indicated antibodies in MCF7. (G) MCF7 cells were cotransfected with HA‐PFKFB3 and Flag‐CHIP plasmid. Western blot analysis tested whole‐cell lysate after 72‐h transfection. (H) MCF7 cells were co‐overexpressed the indicated HA‐CHIP and Flag‐PFKFB3 proteins. Immunoprecipitation with an anti‐Flag antibody was performed. (I) MCF7 cells were co‐overexpressed the indicated HA‐CHIP proteins. Immunoprecipitation with an anti‐PFKFB3 antibody was performed. (J) MCF7 cells were cotransfected with Flag‐PIM2 or sh‐CHIP plasmid. Western blot analysis tested whole‐cell lysate after 72‐h transfection. (K and L) MCF7 cells were cotransfected with Flag‐PIM2 or sh‐PIM2 plasmid. Immunoprecipitation with an anti‐PFKFB3 antibody was performed. (M) MCF7 cells were co‐overexpressed the indicated HA‐PIM2 and Flag‐PFKFB3 (WT, S478A or S478D) proteins. Immunoprecipitation with an anti‐HA antibody was performed. All experiments were repeated at least 3 times

The proteasome pathway is one of the most common protein stability regulatory mechanisms.^21^ In order to study the potential mechanism of PIM2 regulation of PFKFB3 stability, we used MG132 to block protein degradation and found that PFKFB3 degradation was reduced (Figure 4A). We then investigated the ubiquitin levels of PFKFB3 with or without PIM2 expression in MCF‐7 cells. Interestingly, WT PIM2 could reduce the ubiquitination level of PFKFB3 and the opposite occurred when PIM2 was knocked down (Figure 4B and C). Furthermore, PIM2 did not change the ubiquitination level of PFKFB3 S478A (Figure 4D). Previous studies have shown that C‐terminus of Hsc70 interacting protein (CHIP) participates in PIM2‐mediated protein stability regulation.^13^ Likewise, CHIP was interacted with PFKFB3 by mass spectrometry analyses (Figure S1D) and co‐IP (Figures 4E and 4F). Interestingly, CHIP degraded PFKFB3 and increased the ubiquitination level of PFKFB3 (Figures 4G and 4H). Accordingly, we assumed that CHIP and PIM2 similarly regulate the stability of PFKFB3. We further confirmed that the presence of CHIP reduced the interaction of PIM2 and PFKFB3 through co‐IP (Figure 4I). The stability of PFKFB3 was further mediated by PIM2 when CHIP was knocked down (Figure 4J). Similarly, the interaction between PFKFB3 and CHIP was also affected with or without PIM2 (Figures 4K and 4L), and the interaction between CHIP and PFKFB3 S478A was the strongest (Figure 4 M). Together, the findings illustrate that PIM2 promotes PFKFB3 protein stability by CHIP‐mediated ubiquitin‐proteasome pathway.

### 3.5 Phosphorylation of PFKFB3 by PIM2 promotes enzymatic activity and glycolysis in BC

**FIGURE 5:**
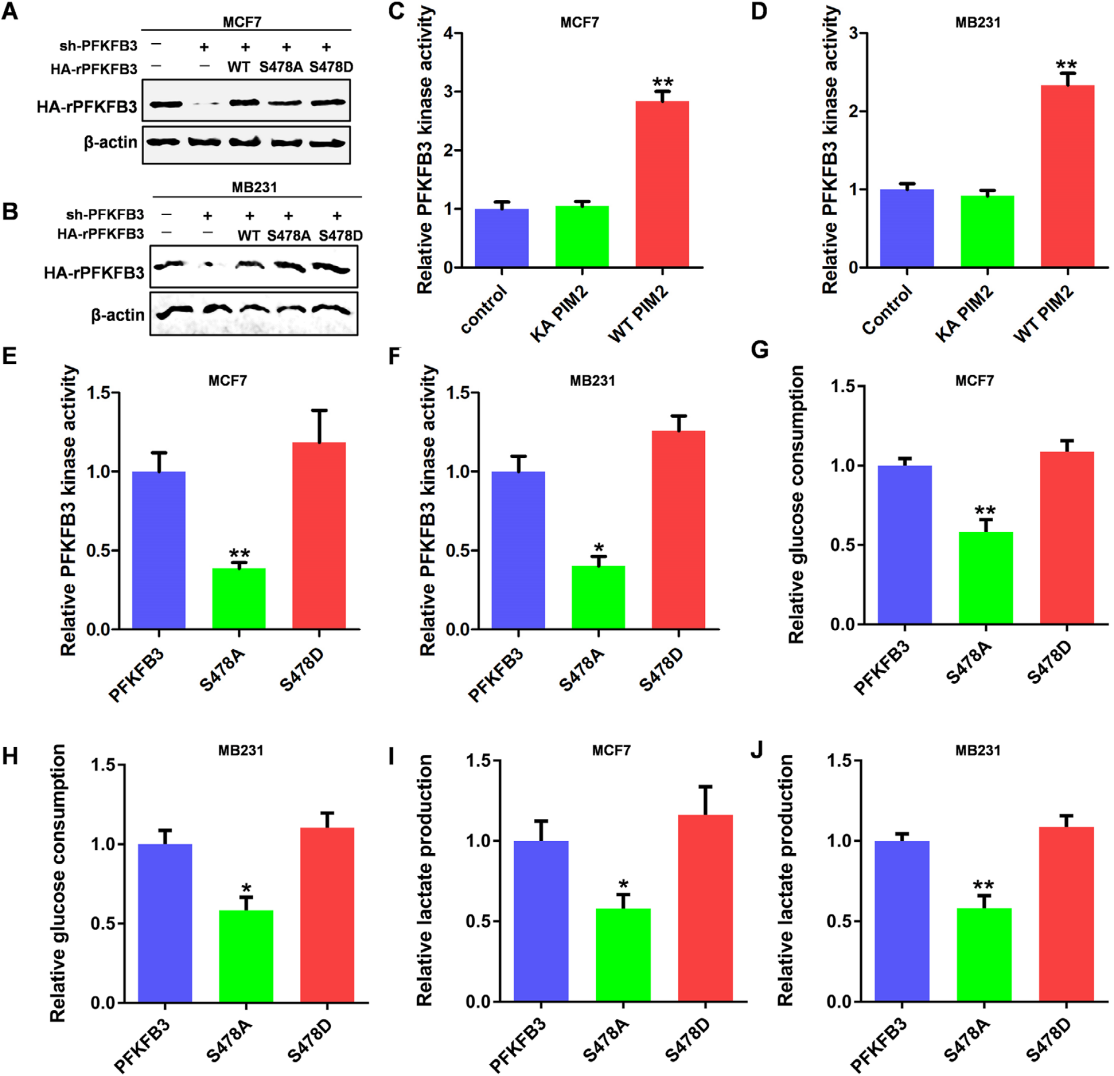
Phosphorylation of PFKFB3 by PIM2 promotes enzymatic activity and glycolysis in BC. (A and B) MCF‐7 and MB231 cells with PFKFB3 depletion reconstituted stable expression of rHAPFKFB3 (WT, S478A, or S478D). Total cell lysates were prepared. (C and D) MCF‐7 and MB231 cells overexpress PIM2 and use the kit to detect the activity of PFKFB3. (E and F) Using the kit to detect the PFKFB3 activity of stable rHAPFKFB3 (WT, S478A, or S478D) cell lines. (G and H) Using the kit to detect the glucose consumption of stable rHAPFKFB3 (WT, S478A, or S478D) cell lines. (I and J) Using the kit to detect the lactate production of stable rHAPFKFB3 (WT, S478A, or S478D) cell lines. All data represent mean ± SD of three independent experiments, *p < 0.05, **p < 0.01

PFKFB3 has a great importance in the regulation of glycolysis, and we have previously confirmed that PIM2 is closely related to glycolysis,^3^, ^22^ thus we hypothesized that PIM2 regulates glycolysis through S478 of PFKFB3. We established stable PFKFB3 shRNA MCF‐7 and MDA‐MB‐231 cells (rPFKFB3) with reconstituted expression of HA‐tagged PFKFB3 (WT, S478A, or S478D) (Figures 5A and 5B). We first evaluated the effect of PIM2 on PFKFB3 kinase activity. As shown in Figure 5C and 5D, PIM2 increased the kinase activity of PFKFB3 in BC cells. The PFKFB3 kinase activity was blocked with PFKFB3 S478A (Figures 5E and 5F). The S478A PFKFB3 mutant stable BC cells decreased both glucose consumption (Figures 5G and 5H) and lactate production (Figures 5I and 5J). These data indicate the phosphorylation of PFKFB3 on S478 plays a role in regulation of its enzymatic activity and glycolysis.

### 3.6 PFKFB3 Ser478 phosphorylation promotes cell proliferation, cell migration, and tumor growth in vivo

**FIGURE 6:**
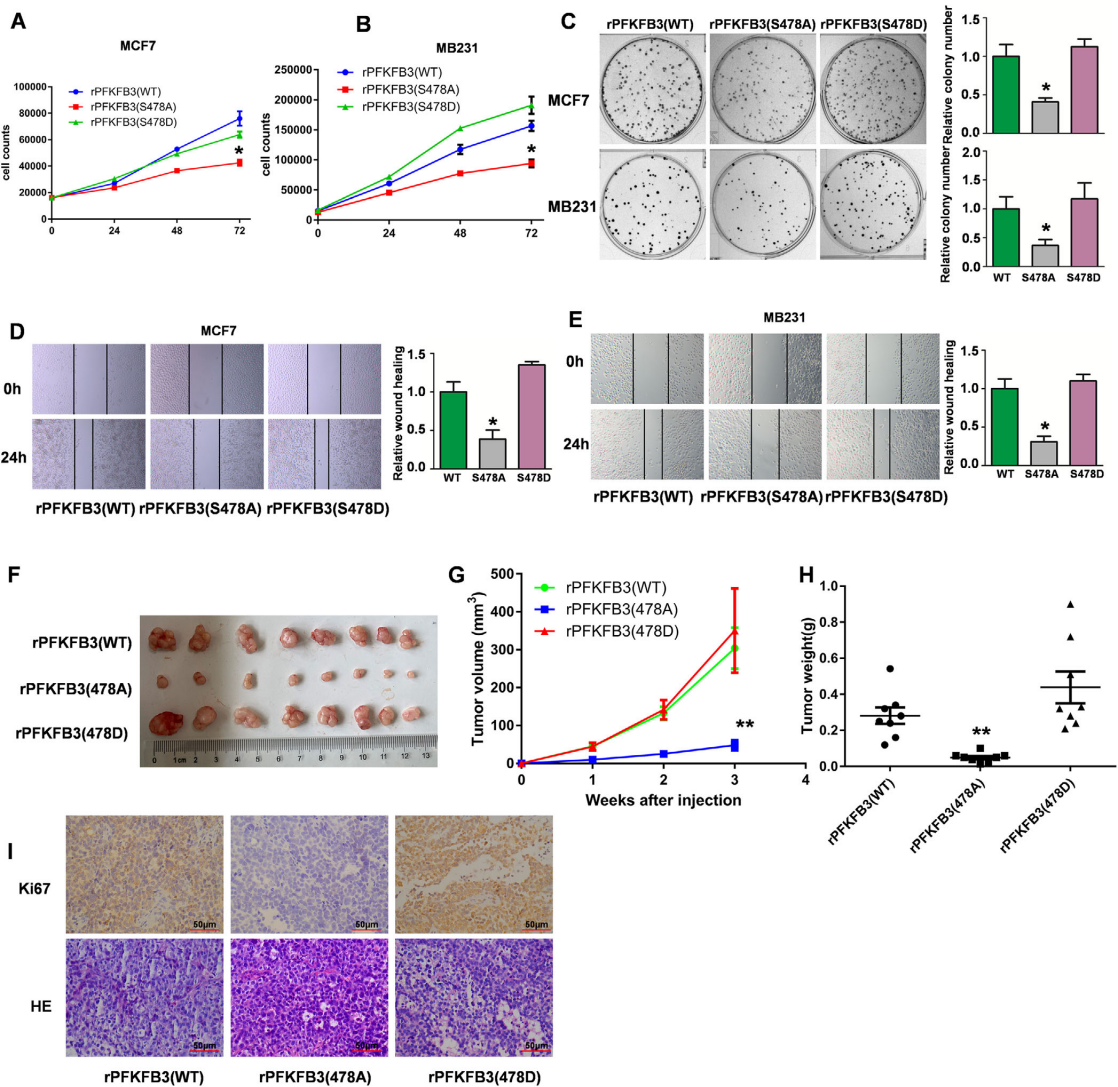
PFKFB3 Ser478 phosphorylation promotes cell proliferation, cell migration, and tumor growth in vivo. (A and B) Cell proliferation of stable rHAPFKFB3 (WT, S478A, or S478D) cell lines. (C) Clone formation of stable rHAPFKFB3 (WT, S478A, or S478D) cell lines. (D and E) Wound healing assay of stable rHAPFKFB3 (WT, S478A, or S478D) cell lines. (F) Tumor formation of stable rHAPFKFB3 (WT, S478A, or S478D) MCF7 cells. (G) Tumor growth of stable rHAPFKFB3 (WT, S478A, or S478D) MCF7 cells. (H) Tumor weight of stable rHAPFKFB3 (WT, S478A, or S478D) MCF7 cells. (I) Ki67 and HE staining of tumor samples. All data represent mean ± SD of three independent experiments, *p < 0.05, **p < 0.01

To clarify the role of PFKFB3 S478 phosphorylation on BC biological significance, we used stable rPFKFB3 BC cells as described above (Figures 5A and 5B). We found that BC cells proliferated much more slowly when rPFKFB3 (S478A) was expressed compared to rPFKFB3 (WT or S478D) (Figure 6A and B), which suggests that PFKFB3 Ser478 phosphorylation promotes cell proliferation. Moreover, PFKFB3 Ser478 phosphorylation by PIM2 enhanced clone formation and migration ability in BC cells, which was determined by clone formation assays and scratch experiments (Figures 6C–6E). To further clarify the effect of PFKFB3 Ser478 phosphorylation on BC, MCF‐7 cells stably expressing rPFKFB3 (WT, S478A, or S478D) were subcutaneously injected into nude mice for the xenograft studies. The results showed that tumor formation ability of rPFKFB3 S478A was much lower than that of the rPFKFB3 WT group or the S478D group (Figure 6F). Consistently, the rPFKFB3 S478A group had a slower tumor growth rate (Figure 6G) and weight (Figure 6H). We also confirmed that cells in rPFKFB3 S478A group were less proliferative than other groups (WT or S478D) in vivo (Figure 6I). All the findings above emphasized the crucial role of phosphorylation of PFKFB3 by PIM2 in BC progression in vivo. These data indicate that PFKFB3 Ser478 phosphorylation promotes cell proliferation, cell migration, and tumor growth in vivo.

### 3.7 Ser478 phosphorylation of PFKFB3 by PIM2 promotes BC cell paclitaxel resistance

**FIGURE 7:**
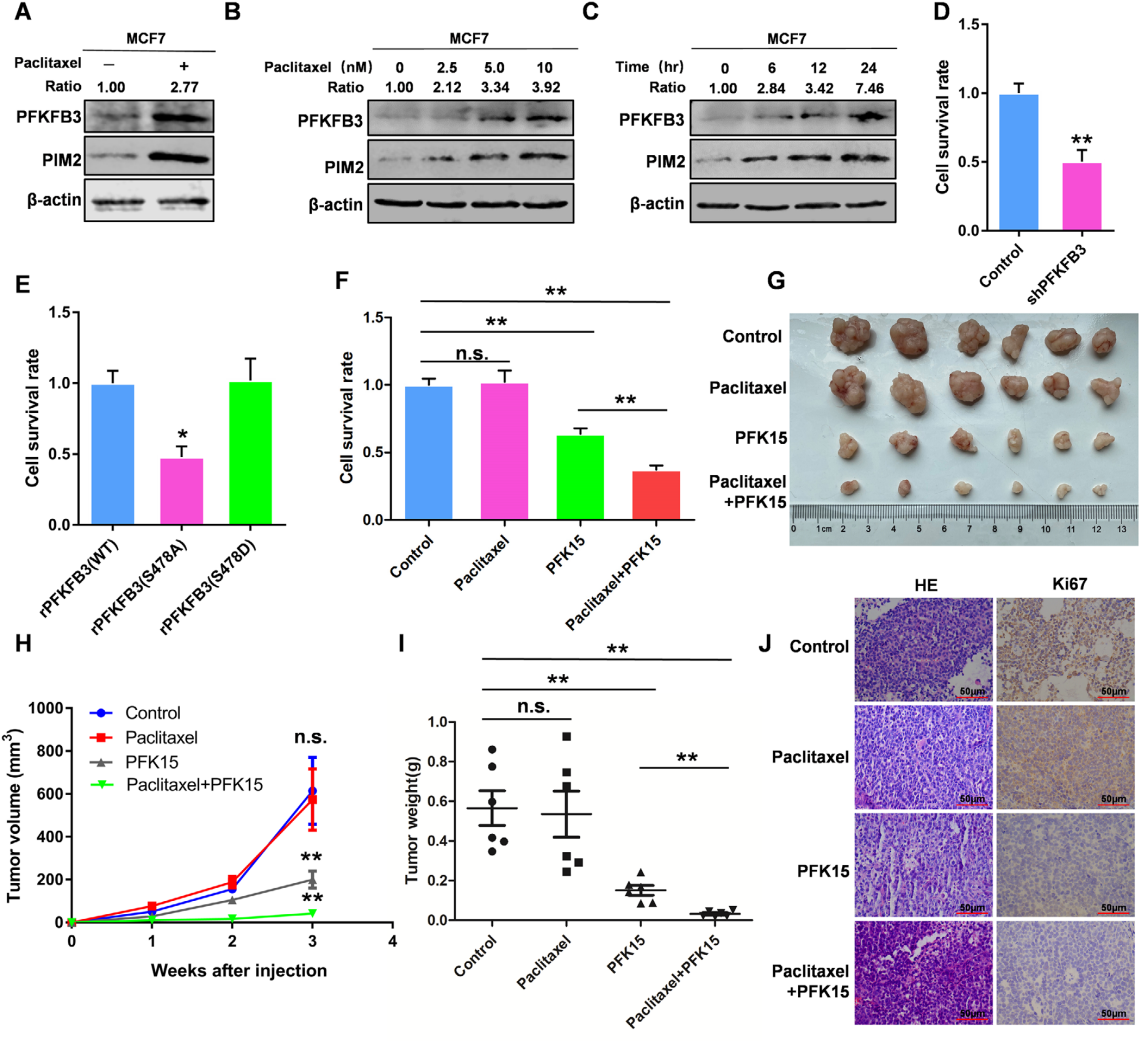
Ser478 phosphorylation of PFKFB3 by PIM2 promotes BC cell paclitaxel resistance. (A) WB detection of PIM2 and PFKFB3 expression in paclitaxel‐resistant strains. (B and C) Treat MCF7 cells with different paclitaxel concentrations or the same concentration with different time, and detect the changes of PIM2 and PFKFB3 by WB. (D) Cell survival rate after knocking out PFKFB3 in paclitaxel‐resistant cell line. (E) Cell survival rate of restore overexpression rHAPFKFB3 (WT, S478A, or S478D) in paclitaxel‐resistant cell line. (F) Cell survival rate of different drug treatment groups (control, paclitaxel, PFK15, paclitaxel+PFK15). (G) Tumor formation of different drug treatment groups (control, paclitaxel, PFK15, paclitaxel+PFK15). (H) Tumor growth of different drug treatment groups (control, paclitaxel, PFK15, paclitaxel+PFK15). (I) Tumor weight of different drug treatment groups (control, paclitaxel, PFK15, paclitaxel+PFK15). (J) Ki67 and HE staining of different drug treatment groups (control, paclitaxel, PFK15, paclitaxel+PFK15). All data represent mean ± SD of three independent experiments, *p < 0.05, **p < 0.01

Chemotherapeutic drug resistance is the primary cause of BC treatment failure.^23^ Chemotherapy, especially paclitaxel, is among the first‐line treatment plans for BC, but it is only effective during the initial stages of treatment. In later stages, the tumor will recur because of the emergence of drug resistance and rapidly reduce patient's life expectancy.^24^ Research shows that increased glycolysis is an important feature of drug‐resistant BC cells. We have previously generated paclitaxel‐resistant BC cell lines (MCF‐7/TaxR) by continuous exposure of the parental drug‐sensitive MCF‐7 cells and confirmed PIM2 expression in MCF‐7/TaxR cells.^3^ Interestingly, we also found the increased expression of PFKFB3 in MCF‐7/TaxR cells (Figure 7A). Moreover, the expression of PIM2 and PFKFB3 was time‐ and dose‐dependent in MCF‐7/TaxR cells (Figures 7B and 7C). To further verify the relationship between PFKFB3 and paclitaxel resistance in BC, we knocked down PFKFB3 in paclitaxel‐resistant MCF‐7 cells and found that the cell proliferation rates decreased (Figure 7D). Surprisingly, compared with MCF‐7 cells expressing rPFKFB3 (WT or S478D), MCF‐7 cells stably expressing rPFKFB3 (S478A) showed decreased resistance to paclitaxel (Figure 7E). We next tested the combined effect of paclitaxel treatment and inhibition of PFKFB3 by PFK15, a potent PFKFB3 inhibitor that could dramatically reduce cell proliferation (Figure 7F). Consistently, compared with the control group, paclitaxel group, and single inhibitor group, combined treatments of paclitaxel and PFK15 were more effective inhibitors of tumor formation (Figure 7G). The combination therapy group also had a much slower tumor growth rate (Figure 7H) and weight (Figure 7I). Ki67 staining illustrated that the combination therapy group inhibited of MCF‐7/TaxR cells (Figure 7J). The findings show that Ser478 phosphorylation of PFKFB3 by PIM2 enhances BC cell resistance to paclitaxel.

### 3.8 PIM2 expression is positively correlated with pS478‐PFKFB3 in BC

**FIGURE 8:**
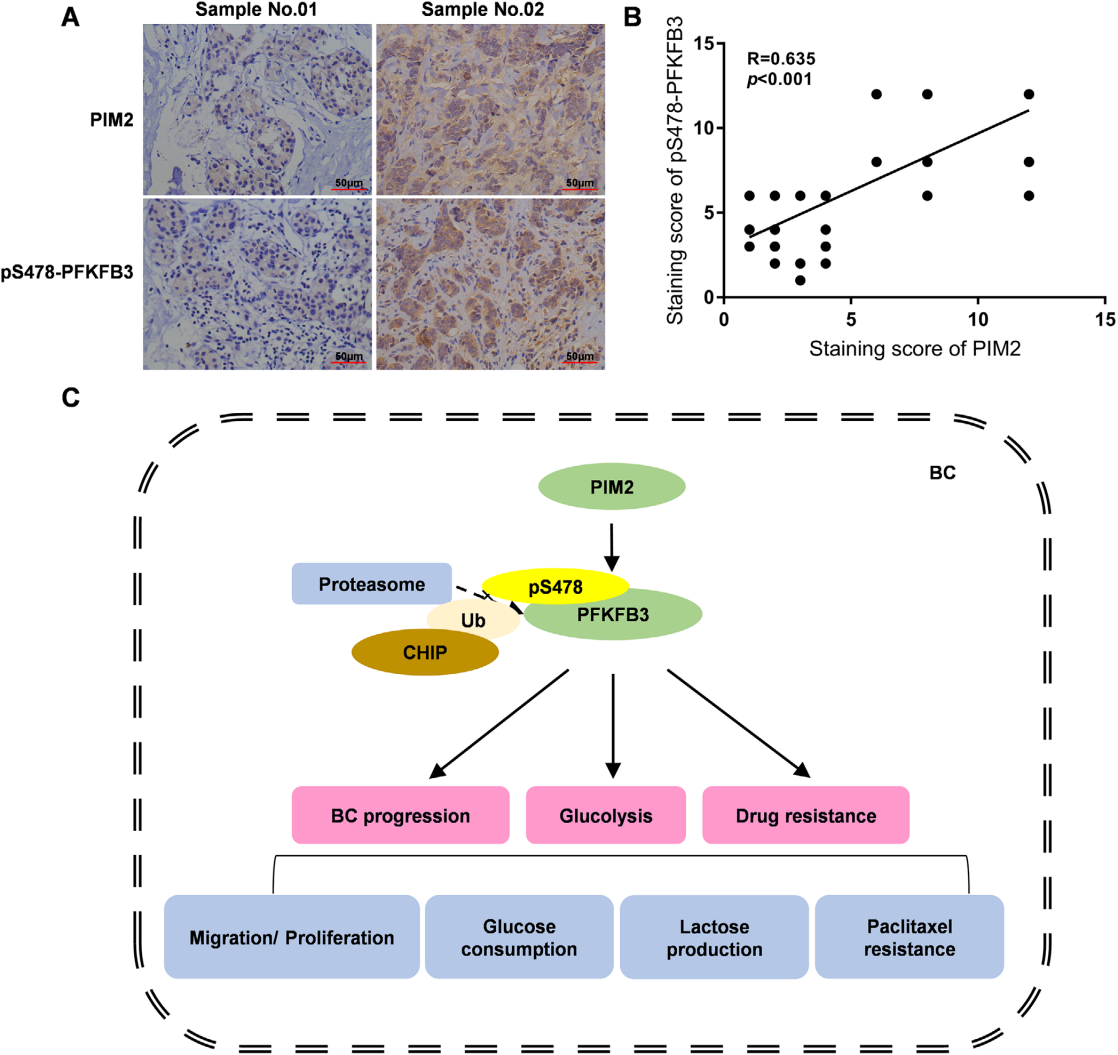
PIM2 expression is positively correlated with pS478‐PFKFB3 in BC. (A) Immunohistochemical expression of PIM2 or pSer478‐PFKFB3 in human BC tissues. (B) Pearson correlation analysis of PIM2 and pSer478‐PFKFB3 semi‐quantitative staining score. (C) A working model that schematic diagram of PIM2 regulating breast cancer glycolysis and paclitaxel resistance through PFKFB3 Ser478 site

Finally, we collected 82 BC samples to test the clinical relationship between the PFKFB3 Ser478 phosphorylation and PIM2 by immunostaining. The details of patient tissues samples were shown in Table S2. As shown in Figure 8A, PFKFB3 Ser478 phosphorylation and PIM2 were strongly expressed in BC samples. Consistent with these data, there was a positive correlation between pS478‐PFKFB3 and PIM2 in human BC tissues (Figure 8B) which also predicted more malignant tumor characteristics.

## 4 DISCUSSION

Cancer cells rewire their metabolism to achieve their infinite proliferation needs.^25^ The "Warburg Effect" is one of the most common and highly researched features.^26^ However, the mechanism of the "Warburg Effect" remains unclear. Here, we analyzed a new mechanism for PFKFB3, the key regulator of glycolysis, in the post‐translational modification and glycolysis regulation in BC. We discovered a novel interaction between PIM2 and PFKFB3 in BC. PIM2 binds to and phosphorylates PFKFB3 at position S478. PIM2 can also promote PFKFB3 protein stability via the CHIP‐mediated ubiquitin‐proteasome pathway. Phosphorylation of PFKFB3 S478 may be involved in regulating aerobic glycolysis, cell proliferation, cell migration, paclitaxel resistance, and tumor growth of BC cells in vivo. Thus, PIM2 may regulate PFKFB3 positively via post‐translational modification.

The first identified rate‐limiting glycolysis step involves 6‐phosphofructose‐1‐kinase (PFK‐1) converting fructose 6‐phosphate (F6P) to fructose‐1,6‐bisphosphate (F1,6P2).^8^, ^9^ PFKFB3 is the strongest allosteric activator of PFK‐1 and plays an indispensable function in regulating glycolysis.^8^ In addition to its glycolytic activities, PFKFB3 also plays an important role in numerous human tumors, including lung, breast, colon, pancreas, ovary, gastric, and glioma tumors.^27^, ^28^, ^29^, ^30^ PFKFB3 stimulates glycolytic flux to support cancer cell growth, and for oncogenic Ras signaling pathway, it is a necessary glycolytic mediator.^31^ Studies have shown that hypoxia can induce transcriptional regulation of PFKFB3.^9^ The estrogen receptor, progesterone receptor, extracellular signal‐regulated kinase, and 5′ AMP‐activated protein kinase can also regulate the transcription of PFKFB3 mRNA in a direct way.^8^, ^32^ Besides the transcriptional control, the reversible post‐translational modification of PFKFB3 can copy with environmental changes more flexibly, economically, and quickly respond to environmental changes at the metabolic level.^8^ The S‐glutathione acylation on Cys206, K142 polyubiquitination and carbon monoxide (CO)‐mediated hypomethylation cause PFKFB3 degradation and shift glucose utilization from glycolysis toward phosphogluconate pathway.^12^, ^33^, ^34^ Here, we identified the phosphorylation of Ser478 of PFKFB3. Importantly, we found that phosphorylation of PFKFB3 S478 has a role in regulating BC glycolysis, cell proliferation, migration, drug resistance, and tumor formation. In addition, other studies have shown that PFKFB3 K472 acetylation and Ser461 phosphorylation can affect the nuclear translocation of PFKFB3.^11^ The Ser478 site is close to Ser461 and K472, and whether its phosphorylation can affect the nuclear translocation of PFKFB3 is unknown and would need further research.

PIM2 belongs to a family of short‐lived serine/threonine kinases that are highly evolutionarily conserved in multicellular organisms.^3^ Numerous studies have shown the overexpression of PIM2 in many cancers, including BC, prostate cancer, endometrial cancer, lung cancer, and lymph cancer.^17^, ^35^, ^36^, ^37^, ^38^ It is known that PIM2 modulates TSC2 phosphorylation and thus maintains multiple myeloma cell growth.^39^ PIM2 promotes hepatocellular carcinoma tumorigenesis and progression through activation of the NF‐κB signaling pathway.^40^ PIM2 promotes tumor progression in many ways; however, its mechanism in glycolysis and tumor resistance is poorly understood. Our data demonstrate that PIM2 can further regulate glucose metabolism and drug resistance by changing the phosphorylation levels of specific PFKFB3 phosphorylation sites, thus showing that PIM2 is important in BC progression.

Metabolic changes in BC not only lead to cancer progression but also lead to drug resistance and drug sensitivity. The increase of energy requirement of drug‐resistant cells was consistent with the enhancement of drug efflux activity and cell detoxification mechanism.^41^ Paclitaxel was a first‐line chemotherapeutic drug often used in BC treatment. Although the initial response to paclitaxel was impressive, most BC patients usually developed resistance, eventually leading to recurrence, metastasis, and death.^42^ Hexokinase 2 was associated with elevated glycolytic flux, which was a characteristic of cancer cells.^24^ Study showed that hexokinase 2 was overexpressed and activated in BC, and it could help to increase the paclitaxel resistance.^3^ lactate dehydrogenase A (LDHA) was a key glycolytic enzyme during the conversion from pyruvate to lactate. The downregulation of LDHA by siRNA significantly increased the sensitivity of Taxol‐resistant cells to Taxol in BC.^43^ In our study, we found a new mechanism of paclitaxel resistance in BC. PIM2 could reduce the paclitaxel sensitivity by changing the activity of PFKFB3 in BC.

In summary, we identified that PIM2 is a new binding protein of PFKFB3. We used biochemical methods to determine that PIM2 can directly bind and change the phosphorylation of PFKFB3 at Ser478 to enhance PFKFB3 protein stability through the ubiquitin‐proteasome pathway (Figure 8C). Importantly, in vitro and in vivo, phosphorylation of PFKFB3 at Ser478 promoted glycolysis, BC cell growth, and paclitaxel resistance together with PIM2. Therefore, our research clarifies the regulation of BC glucose metabolism and provides theoretical support for new methods of targeted diagnosis and treatment of tumors.

## CONFLICT OF INTEREST

The authors declare that they have no conflict of interest.

## AUTHOR CONTRIBUTIONS

Zhenhai Yu, Chune Ren, and Chao Lu designed research. Chao Lu, Chune Ren, Pengyun Qiao, and Yonghong Sun performed research. Chao Lu and Yonghong Sun contributed new reagents/analytic tools. Zhenhai Yu, Chao Lu, and Chune Ren analyzed data. Chao Lu wrote and revised the paper.

## Supporting information

Table S1: Primer list

**Figure S1** (A) Confocal immunofluorescence microscopy was performed to analyze localization of PIM2 and PFKFB3 in MCF‐7 cells. (B and C) MCF7 or MB231 cells were knocked down PIM2 with shRNA. Confocal immunofluorescence microscopy was performed to observe the expression of PIM2 and PFKFB3. (D) Mass spectrometry analyses of the immunoprecipitated PFKFB3 complex in MCF‐7 cells

Supporting Information

## Data Availability

Wiley have a bold vision for the future in which research data is shared openly, and we plan to accomplish this vision through partnerships and collaborations. We currently partner with Dryad to make it easy for authors to share data in approved repositories to comply with funder and journal mandates.
