# Supplementary material for: Positive regulation of PFKFB3 by PIM2 promotes glycolysis and paclitaxel resistance in breast cancer
Source: Clin Transl Med. 2021 May 1;11(4):e400. doi: 10.1002/ctm2.400 (PMC8087946; doi:10.1002/ctm2.400)
Supplement: Supplementary file 1 — Table S1: Primer list [file CTM2-11-e400-s001.doc]

**Table S1: Primer list**

| Gene | Sequence (5’ to 3’) |
| --- | --- |
| shNC | TTCTCCGAACGGTCACGT |
| shPFKFB3 | CGTGTCGGTTCCATTCCATTT |
| shPIM2 | CTCGAAGTCGCACTGCTAT |
| shCHIP | TTACACCAACCGGGCCTTG |

**Table S2: The details of patient tissues samples**

| Number | n |
| --- | --- |
| Age |  |
| ≤50y | 32 |
| >50y | 50 |
| Tumor size |  |
| ≤2 cm | 36 |
| >2 cm | 46 |
| TNM stage |  |
| Ⅰ-Ⅱ | 39 |
| Ⅲ-Ⅳ | 43 |
| ER status |  |
| + | 49 |
| – | 33 |
| PR status |  |
| + | 51 |
| – | 31 |
| HER2 |  |
| + | 37 |
| – | 45 |
