# Supplementary figures and images for: Positive regulation of PFKFB3 by PIM2 promotes glycolysis and paclitaxel resistance in breast cancer
Source: Clin Transl Med. 2021 May 1;11(4):e400. doi: 10.1002/ctm2.400 (PMC8087946; doi:10.1002/ctm2.400)

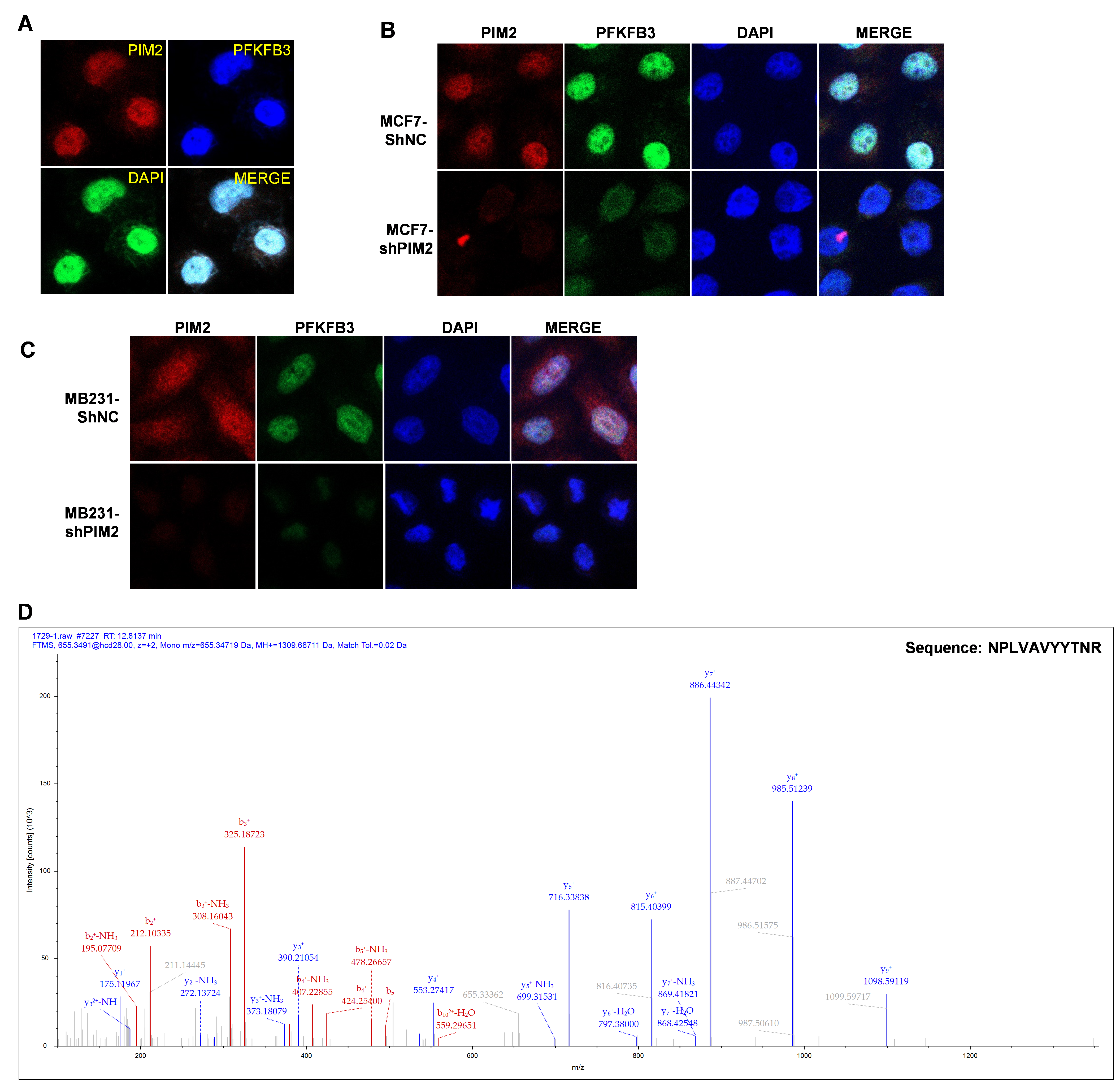

Supplement: Supplementary file 2 — Figure S1 (A) Confocal immunofluorescence microscopy was performed to analyze localization of PIM2 and PFKFB3 in MCF‐7 cells. (B and C) MCF7 or MB231 cells were knocked down PIM2 with shRNA. Confocal immunofluorescence microscopy was performed to observe the expression of PIM2 and PFKFB3. (D) Mass spectrometry analyses of the immunoprecipitated PFKFB3 complex in MCF‐7 cells [file CTM2-11-e400-s003.tif]
